# Recurrence of common bile duct stones after choledocholithotomy in elderly patients: risk factor analysis and clinical prediction model development

**DOI:** 10.3389/fmed.2023.1239902

**Published:** 2023-10-23

**Authors:** Han Wang, Yu-qi He, Shi-yang Dong, Wan Zhong, Ping Tao, Shi-yong Yang, Zi-jun Liu

**Affiliations:** Department of General Surgery, Nanjing First Hospital, Nanjing Medical University, Nanjing, Jiangsu, China

**Keywords:** common bile duct stones, recurrence, choledocholithotomy, risk factors, prediction model

## Abstract

**Background:**

The reasons for the recurrence of common bile duct stones (CBDS) in elderly patients after choledocholithotomy are still unclear. This study aims to establish a prediction model for CBDS recurrence by identifying risk factors.

**Methods:**

We conducted a retrospective analysis of 1804 elderly patients aged 65 years and above who were diagnosed to have CBDS and were admitted to Nanjing First Hospital between January 1, 2010, and January 1, 2021. According to inclusion and exclusion criteria, 706 patients were selected for the final analysis. The patients were assigned to two groups according to the presence or absence of CBDS recurrence, and their clinical data were then statistically analyzed. Subsequently, a prediction model and nomogram were developed, evaluating effectiveness using the concordance index (C-index).

**Results:**

Of the 706 elderly patients, 62 patients experienced CBDS recurrence after surgery, resulting in a recurrence rate of 8.8%. The multivariate Cox analysis showed that prior history of cholecystectomy (hazard ratio [HR] = 1.931, 95% confidence interval [CI]: 1.051–3.547, *p* = 0.034), white blood cell (WBC) count ≥11.0 × 10^9^/L (HR = 2.923, 95% CI: 1.723–4.957, *p* < 0.001), preoperative total bilirubin (TBIL) level ≥ 36.5 mmol/L (HR = 2.172, 95% CI: 1.296–3.639, *p* = 0.003), number of stones ≥2 (HR = 2.093, 95% CI: 1.592–5.294, *p* = 0.001), maximum stone diameter ≥ 0.85 cm (HR = 1.940, 95% CI: 1.090–3.452, *p* = 0.024), and T-tube drainage (HR = 2.718, 95% CI: 1.230–6.010, *p* = 0.013) were independent risk factors of CBDS recurrence in elderly patients after choledocholithotomy. A postoperative CBDS recurrence prediction model was constructed with a C-index value of 0.758 (95% CI: 0.698–0.818) and internal validation value of 0.758 (95% CI: 0.641–0.875).

**Conclusion:**

A history of cholecystectomy, WBC count ≥11.0 × 10^9^/L, preoperative TBIL level ≥ 36.5 mmol/L, number of stones ≥2, maximum stone diameter ≥ 0.85 cm, and T-tube drainage are the independent risk factors of CBDS recurrence after choledocholithotomy in elderly patients. Our developed prediction model for CBDS recurrence has good predictive ability and can help predict the prognosis of patients with CBDS.

## 1. Introduction

Common bile duct stones (CBDS) are a prevalent digestive disorder with a high incidence. It is a chronic recurrent hepatobiliary disease that develops due to impaired metabolism of cholesterol, bilirubin, and bile acid (1). The incidence of cholelithiasis is 5–15%, among which the incidence of CBDS is approximately 5–30% (2). It is known that CBDS incidence increases with age, particularly in the elderly population (3). CBDS occur when a gallstone blocks the common bile duct (CBD), thereby blocking the passage of the bile, which flows back into the liver (4). This can lead to symptoms such as right-sided abdominal pain, jaundice, nausea, vomiting, and fever. If the stone is deposited in the CBD and blocks it, acute obstructive suppurative cholangitis can occur, which causes symptoms such as septic shock and altered consciousness and is a life-threatening condition, particularly in elderly patients (5).

Presently, the optimal treatment for CBDS remains controversial. The most widely accepted techniques are endoscopic retrograde cholangiopancreatography (ERCP) plus laparoscopic cholecystectomy (LC) and laparoscopic common bile duct exploration (LCBDE) (3). According to previous studies, LCBDE and ERCP have similar postoperative complication and mortality rates in the general population; however, LCBDE has certain advantages over ERCP with regard to the stone clearance rate, overall success rate, and length of hospital stay, which make the former more cost-effective (6–9). Following advances in medical technology, the success rate of treating CBDS has greatly increased. However, even after the complete removal of stones, CBDS recurrence is a common phenomenon, ranging from approximately 4–25%, with the recurrence rate increasing over time (10–13). According to literature reports, the risk factors for CBDS recurrence mainly include age, lipid metabolism, nutrition, obesity, biliary infection, bile stasis, biliary inflammation, periampullary diverticulum, the number and size of stones, CBD diameter, and history of previous cholecystectomy or ERCP (2, 14, 15). These risk factors, however, remain debatable, and the extent to which each factor influences CBDS recurrence and their interplay remain unclear. Consequently, based on these research findings, clinicians are unable to assess the likelihood of postoperative CBDS recurrence in patients, thus making it difficult to implement early prevention strategies for high-risk populations.

In this context, we analyzed the clinical data of patients to identify the most appropriate predictive factors and subsequently constructed a prediction model for CBDS recurrence after choledocholithotomy in elderly patients.

## 2. Materials and methods

### 2.1. Inclusion and exclusion criteria

We conducted a retrospective analysis of the clinical data of patients who were diagnosed to have CBDS and were admitted to Nanjing First Hospital from January 1, 2010, to January 1, 2021. Our study included elderly patients aged 65 years and above. The following exclusion criteria were considered: (a) patients who did not undergo choledocholithotomy during hospitalization; (b) patients with concurrent intrahepatic bile duct stones; (c) patients with a history of tumors in the bile duct, pancreas, duodenal papilla, or other organs; and (d) patients with missing medical records or were lost to follow-up. Finally, 706 patients were included in the study and were assigned to two groups based on the presence or absence of CBDS recurrence for data analysis. The study was conducted in accordance with the Helsinki Declaration and was approved by the ethics committee of Nanjing First Hospital.

### 2.2. Endpoints

The primary endpoints were the occurrence rate and risk factors of CBDS recurrence. The recurrence of CBDS was defined as 6 months after the complete resection of primary stones (16, 17).

### 2.3. Data collection

The following patient-related data were retrieved from the hospital information system:

- Basic information: hospitalization number; gender; age; and history of cardiovascular disease, hypertension, diabetes, pulmonary disease, cholecystectomy, and ERCP.
- Preoperative indices: CBD diameter, number of stones, maximum stone diameter, white blood cell count (WBC), hemoglobin level, serum alanine aminotransferase (ALT), serum aspartate aminotransferase (AST), total bilirubin (TBIL), alkaline phosphatase (ALP), γ-glutamyl transferase (GGT), and total bile acid.
- Hospitalization information: surgical procedure (LCBDE or open common bile duct exploration [OCBDE]), CBD closure method (primary closure or T-tube drainage), postoperative complications (hemorrhage, biliary fistula, and abdominal infection), and postoperative hospital stay.

### 2.4. Follow up data

We collected follow-up data from the medical records of CBDS patients after surgery, including readmission records; findings of ultrasound, CT, or MRI scans; and results of other diagnostic tests. We also obtained information about the patients’ postoperative conditions through telephonic consultations and outpatient visits to determine the presence of stone recurrence and the timing of recurrence.

### 2.5. Statistical methods

All data were statistically analyzed and processed using SPSS 26.0 and R Studio. Continuous variables with normal distribution were expressed as mean ± standard deviation and compared between both groups using t-test. Continuous variables with non-normal distribution were reported as median (M) (P25, P75) and compared between both groups by using Mann–Whitney *U* test. Categorical variables were expressed as counts (percentages) and compared between both groups by using the chi-square test or Fisher’s exact probability test. The Kaplan–Meier method was used to construct survival curves, and log-rank tests were used to compare the survival curves of the recurrence and non-recurrence groups. Cox regression models were used for multivariate survival analysis. The *p* < 0.05 was considered statistical significance. Finally, following consultation with clinicians, the best predictors were identified and used to construct a prediction model. The model’s discriminative ability and accuracy were evaluated using the concordance index (C-index) and a calibration curve based on internal validation, respectively.

## 3. Results

### 3.1. Characteristics of included patients

**Figure 1:**
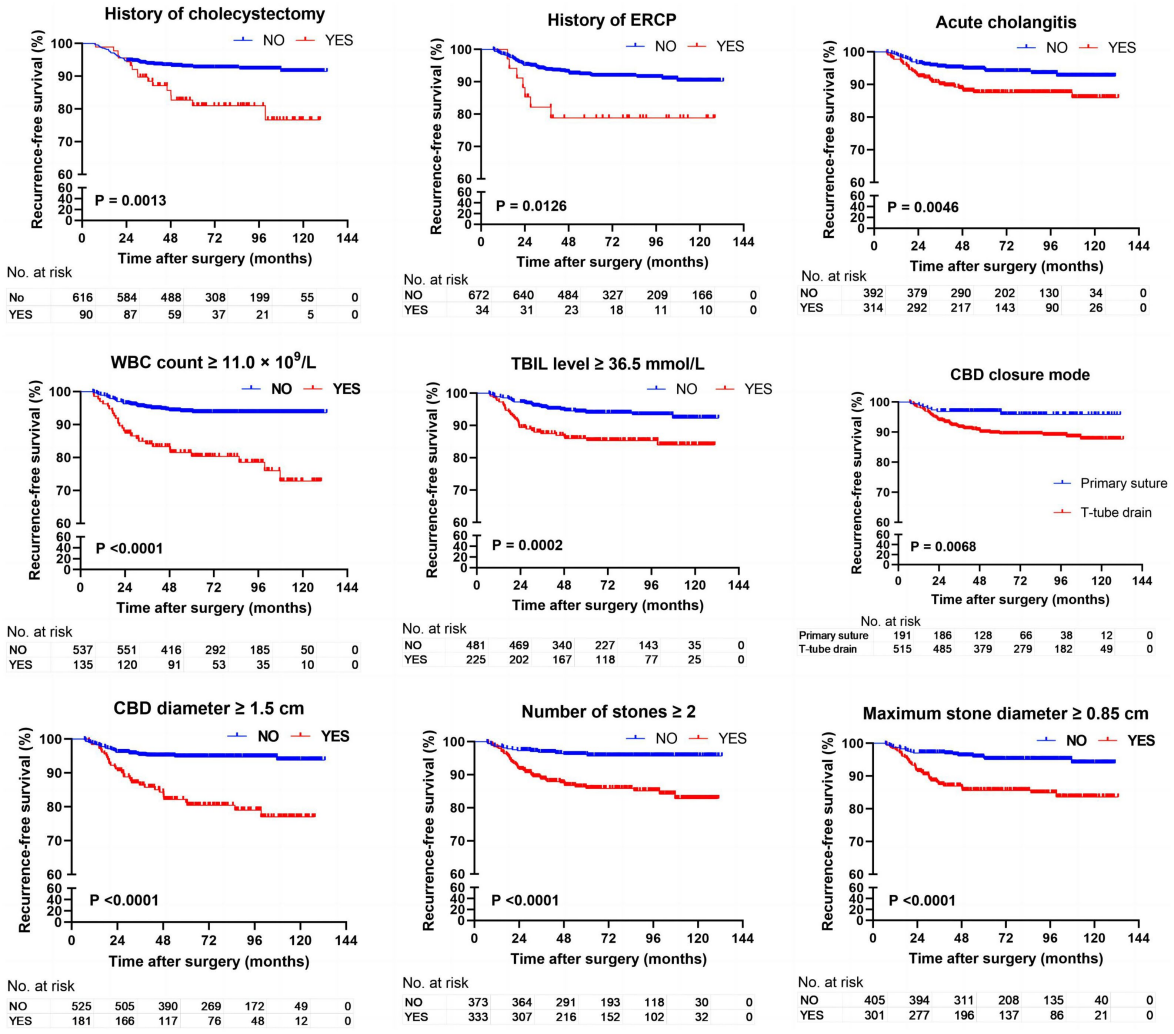
Kaplan–Meier analysis comparing the impact of various influencing factors on the recurrence free survival rate of CBDS.

A total of 706 patients (212 cases of laparoscopic surgery and 494 cases of open surgery) were included in the study, and they were divided into two groups for data analysis based on the occurrence of CBDS recurrence. The recurrence and non-recurrence groups showed no significant differences with regard to age, gender, and chronic comorbidities (cardiovascular disease, hypertension, diabetes, and pulmonary disease). Compared to the non-recurrence group, the recurrence group had a significantly higher proportion of patients with a history of cholecystectomy, ERCP, and acute cholangitis; number of stones ≥2; and placement of a T-tube drainage catheter in the bile duct (*p* < 0.05). Patients in the recurrence group also had a significantly higher preoperative WBC count, preoperative TBIL level, and CBD diameter and maximum stone diameter (Table 1). A noteworthy finding was that the recurrence rates showed no significant difference between the two surgical methods (OCBDE vs. LCBDE). These results were further confirmed by the Kaplan–Meier method and the log-rank test (Figure 1).

**Table 1:** Characteristics of the study patients.

Factors	Recurrence group (*n* = 62)	Non-recurrence group (*n* = 644)	*p*-value
Sex, *n* (%)			0.877
Male	32 (51.6%)	339 (52.6%)	
Female	30 (48.4%)	305 (47.4%)	
Age	76 (71, 82)	75 (70, 81)	0.417
Hypertension, *n* (%)	33 (53.2%)	360 (55.9%)	0.686
Diabetes, *n* (%)	8 (12.9%)	124 (19.3%)	0.221
Angiocardiopathy, *n* (%)	16 (25.8%)	152 (23.6%)	0.697
Pulmonary disease, *n* (%)	6 (9.7%)	50 (7.8%)	0.775
History of cholecystectomy, *n* (%)	16 (25.8%)	74 (11.5%)	0.001
History of ERCP, *n* (%)	7 (11.3%)	27 (4.2%)	0.029
Acute cholangitis, *n* (%)	38 (61.3%)	276 (42.9%)	0.005
WBC (×10^9^/L)	10.5 (5.7, 14.2)	7.9 (5, 9.3)	<0.001
HB (g/L)	120 (111, 132)	123 (114, 133)	0.252
ALT (U/L)	116 (37, 143)	126 (23, 166)	0.655
AST (U/L)	99 (24, 114)	109 (22, 129)	0.669
TBIL (g/L)	53.0 (17.4, 86.5)	38.7 (12.4, 88.2)	0.001
ALP (U/L)	318 (103, 409)	319 (84, 664)	0.915
GGT (U/L)	189 (97, 235)	205 (92, 385)	0.605
Total bile acid (μmol/L)	41 (3, 190)	60 (4, 212)	0.186
Serum creatinine, (μmol/L)	72 (56, 103)	75 (59, 102)	0.393
CBD diameter (cm)	1.5 (1.2, 1.7)	1.2 (0.9, 1.4)	<0.001
Number of stones ≥2, *n* (%)	47 (75.8%)	286 (44.4%)	<0.001
Maximum stone diameter (cm)	1.06 (0.7, 1.2)	0.84 (0.5, 1.4)	<0.001
CBD closure mode, *n* (%)			0.003
T-tube drain	55 (88.7%)	460 (71.4%)	
Primary suture	7 (11.3%)	184 (28.6%)	
Surgical modalities, *n* (%)			0.055
LCBDE	12 (19.4%)	200 (31.3%)	
OCBDE	50 (80.6%)	444 (68.9%)	
Postoperative complications, *n* (%)	5 (8.1%)	33 (5.1%)	0.493
Hospital stay (days)	11.2 (7, 12)	9.8 (7, 11)	0.417

### 3.2. Univariate and multivariate Cox analyses of CBDS recurrence after choledocholithotomy in elderly patients

A univariate Cox analysis was conducted on all factors with CBDS recurrence and time to recurrence as dependent variables. The results showed significant differences (*p* < 0.05) in the two groups with regard to the following factors: a history of cholecystectomy, ERCP, and acute cholangitis; preoperative WBC count; preoperative TBIL level; CBD diameter; number of stones ≥2; maximum stone diameter; and CBD closure method. We then used receiver operating characteristic (ROC) curve analysis to determine the cut-off values for continuous variables, converted them into categorical variables. The final cut-off values selected were as follows: WBC count: 11 × 10^9^/L; preoperative TBIL level: 36.5 mmol/L; CBD diameter: 1.5 cm; and maximum stone diameter: 0.85 cm. Finally, 9 significant factors (*p* < 0.05) identified in the univariate analysis were included in the multivariate Cox analysis. The results showed that prior history of cholecystectomy (hazard ratio [HR] = 1.931, 95% confidence interval [CI]: 1.051–3.547, *p* = 0.034), WBC count ≥11.0 × 10^9^/L (HR = 2.923, 95% CI: 1.723–4.957, *p* < 0.001), preoperative TBIL level ≥ 36.5 mmol/L (HR = 2.172, 95% CI: 1.296–3.639, *p* = 0.003), number of stones ≥2 (HR = 2.093, 95% CI: 1.592–5.294, *p* = 0.001), maximum stone diameter ≥ 0.85 cm (HR = 1.940, 95% CI: 1.090–3.452, *p* = 0.024), and T-tube drainage (HR = 2.718, 95% CI: 1.230–6.010, *p* = 0.013) were the independent risk factors for CBDS recurrence after choledocholithotomy in elderly patients (Table 2).

**Table 2:** Univariate and multivariate Cox analyses of factors affecting CBDS recurrence.

Factors	Univariate analysis			Multivariate analysis		
	HR	95% CI	*p*-value	HR	95% CI	*p*-value
Sex	0.969	0.589–1.595	0.901			
Age	1.018	0.982–1.056	0.334			
Hypertension	0.922	0.560–1.519	0.750			
Diabetes	0.633	0.301–1.330	0.227			
Angiocardiopathy	1.128	0.638–1.992	0.679			
Pulmonary disease	1.298	0.559–3.013	0.554			
History of cholecystectomy	2.473	1.400–4.369	0.001^*^	1.931	1.051–3.547	0.034^*^
History of ERCP	2.621	1.194–5.757	0.013^*^	1.637	0.709–3.775	0.248
Acute cholangitis	2.062	1.237–3.438	0.005^*^	1.320	0.773–2.256	0.309
WBC	1.087	1.047–1.128	<0.001^*^	2.923	1.723–4.957	<0.001^*^
Hemoglobin	0.990	0.975–1.005	0.188			
ALT	0.999	0.998–1.001	0.577			
AST	1.000	0.998–1.001	0.594			
TBIL	1.004	1.000–1.008	<0.001^*^	2.172	1.296–3.639	0.003^*^
ALP	1.000	0.999–1.001	0.976			
GGT	1.000	0.998–1.001	0.528			
Total bile acid	0.997	0.994–1.001	0.129			
Serum creatinine	0.996	0.985–1.006	0.394			
CBD diameter	3.458	2.304–5.190	<0.001^*^	1.655	0.946–2.893	0.077
Number of stones ≥2	3.727	2.084–6.666	<0.001^*^	2.093	1.592–5.294	0.001^*^
Maximum stone diameter	1.156	1.188–1.936	<0.001^*^	1.940	1.090–3.452	0.024^*^
CBD closure mode	2.830	1.288–6.218	0.007^*^	2.718	1.230–6.010	0.013^*^
Surgical modalities	0.646	0.343–1.218	0.177			
Postoperative complications	1.673	0.671–4.175	0.270			
Hospital stay	1.039	1.003–1.075	0.131			

*Indicated statistical significance.

### 3.3. Construction and evaluation of the prediction model

**Figure 2:**
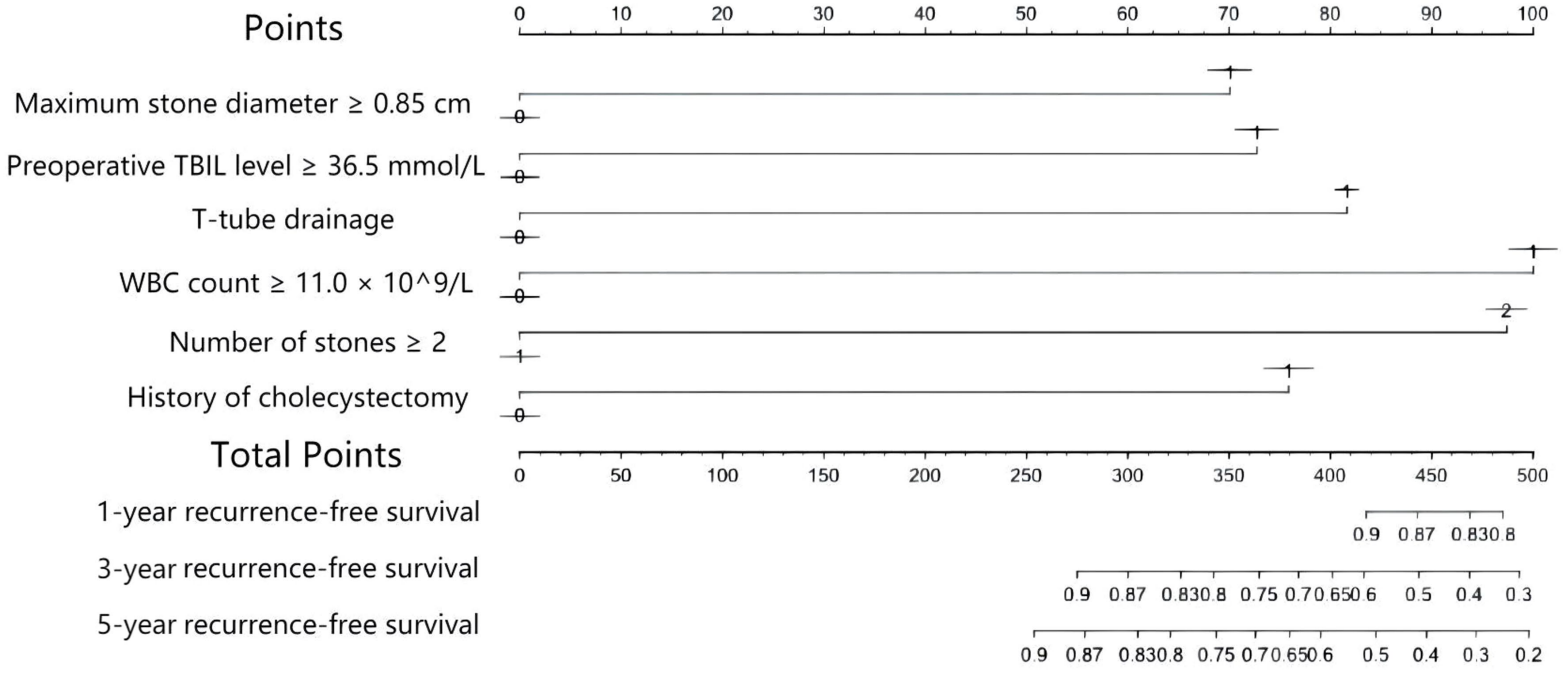
The nomogram to construct a predictive model of CBDS recurrence.

**Figure 3:**
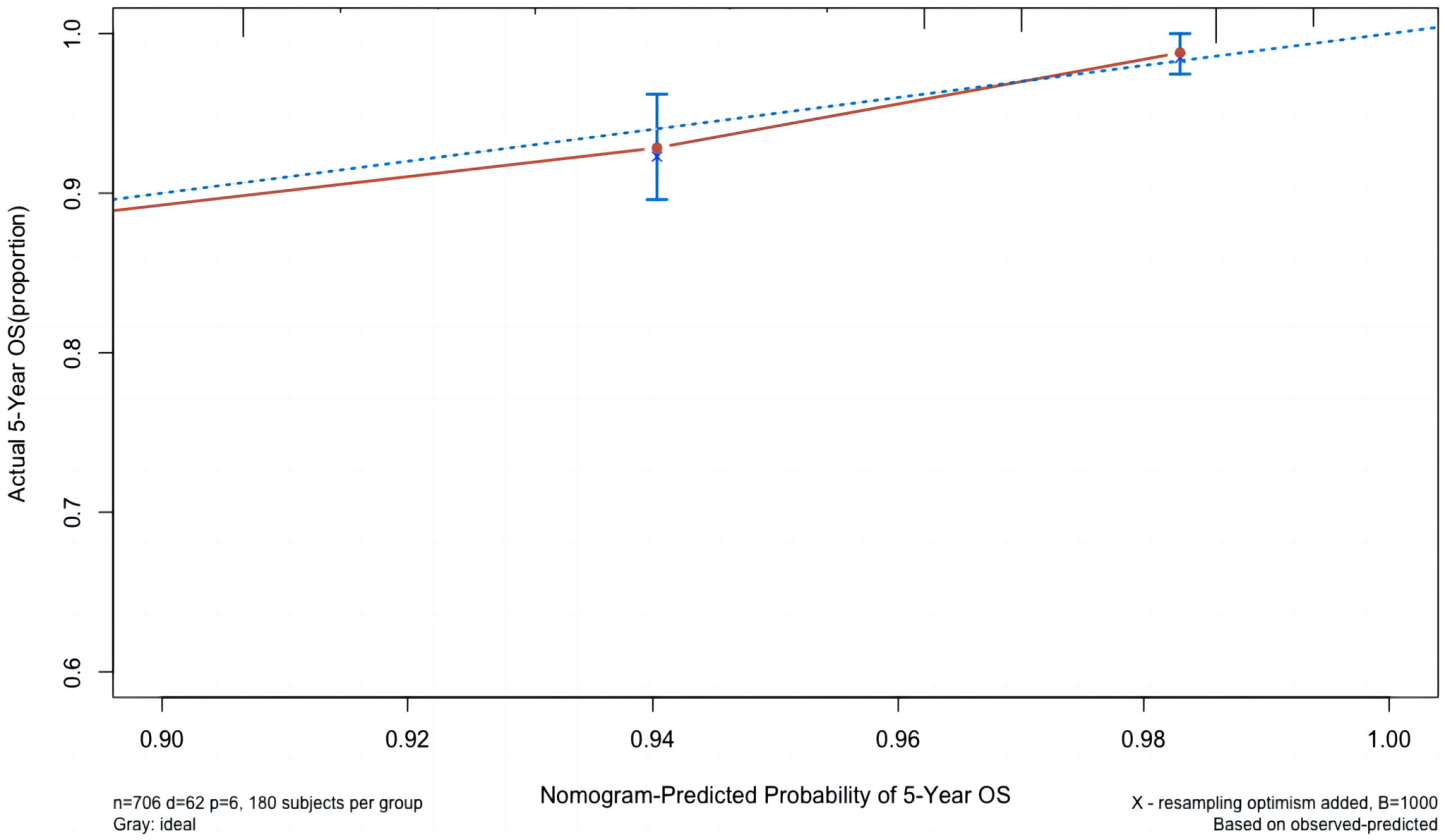
The calibration curve of the nomogram model.

Based on the results of the multivariate Cox analysis and suggestions of clinicians, five factors, i.e., a history of cholecystectomy, WBC count, number of stones, maximum stone diameter, and CBD closure mode, were included in the Cox regression model. A nomogram to predict CBDS recurrence at 1, 3, and 5 years after choledocholithotomy was constructed (Figure 2). In this nomogram, the predicted probability of CBDS recurrence was mapped to a range from 0 to 400. For each variable, a vertical line was drawn upward, and the intersection was marked on the single score axis to indicate its individual score. The sum of the individual scores of the five variables represented the total score of the patient, which corresponded to the probability of no CBDS recurrence at 1, 3, and 5 years on the prediction nomogram. The CBDS recurrence probability was 1 minus the probability of no CBDS recurrence. The nomogram model’s performance for differentiation and accuracy was validated based on the C-index and a calibration curve. The C-index was 0.758 (95% CI: 0.698–0.818), and the internal validation value determined by the bootstrap method with 1,000 times resampling was 0.758 (95% CI: 0.641–0.875). The calibration curves with 1,000 bootstrapping replications (Figure 3) showed a good agreement between the predicted and observed results.

## 4. Discussion

CBDS is a common disease for elderly patients (18). The natural history of CBDS is less well known than that of gall bladder stones. Complications of CBDS include pain, partial or complete biliary obstruction leading to obstructive jaundice, cholangitis, hepatic abscesses, pancreatitis, and secondary biliary cirrhosis, which are potentially life-threatening (19). Patients diagnosed to have CBDS should be recommended to undergo extraction of stones whenever possible, as it provides significant benefits, particularly for symptomatic patients (19–21).

CBDS recurrence poses a significant challenge for physicians. In our study, approximately 8.8% of patients had CBDS recurrence after stone removal procedures and surgeries; this rate was similar to that reported in previous studies (13, 22). A history of cholecystectomy, WBC count ≥11.0 × 10^9^/L, preoperative TBIL level ≥ 36.5 mmol/L, number of stones ≥2, maximum stone diameter ≥ 0.85 cm, and T-tube drainage were independent risk factors affecting CBDS recurrence in elderly patients after choledocholithotomy. A history of cholecystectomy and the placement of a T-tube drainage catheter are independent risk factors as compared to other factors and have been rarely reported as significant factors of CBDS recurrence in previous studies (2, 13, 23).

It remains controversial whether a history of cholecystectomy is associated with CBDS recurrence. Because some CBDS are formed due to the migration of gallbladder stones, also known as secondary CBDS, it is generally believed that cholecystectomy can prevent CBDS recurrence to some extent (24). However, Heo et al. (25) argued that cholecystectomy was not correlated with a reduction in CBDS recurrence. Furthermore, a case–control study showed no significant difference in the cumulative CBDS recurrence rate between the cholecystectomy group and the non-cholecystectomy group (16). In contrast, other researchers believed that a history of cholecystectomy was closely associated with CBDS recurrence (13). In our present study, patients with a history of cholecystectomy had a significantly higher rate of CBDS recurrence than those without cholecystectomy (17.78% vs. 7.47%, *p* < 0.001). Furthermore, the multivariate Cox analysis revealed that a history of cholecystectomy was an independent risk factor for CBDS recurrence after choledocholithotomy in elderly patients. This may be related to post-cholecystectomy dysfunction of the Oddi sphincter. After cholecystectomy, the damage to the nerves surrounding the gallbladder neck and the abnormal secretion of cholecystokinin can lead to a spasm of the Oddi sphincter, ultimately resulting in biliary stasis and CBDS recurrence (26–29). Intraoperative manipulation and postoperative adhesions can also potentially cause inadvertent bending or narrowing of the CBD, which is also a risk factor for promoting the formation of CBDS (30).

The placement of a T-tube drainage catheter in the biliary tract is also a debatable topic. Our multivariate analysis revealed that CBDS recurrence was 2.7-fold higher in patients with T-tube insertion than in those with no T-tube insertion (HR = 2.718, 95% CI: 1.230–6.010, *p* = 0.013). Improper placement of the T-tube can lead to the twisting or bending of the CBD, while prolonged T-tube drainage can increase the risk of bacterial infections within the bile duct, which may contribute to the high recurrence rate of CBDS. It is worth noting that when surgeons suspect the potential recurrence of CBDS after surgery, they often choose to place a T-tube, which may lead to selection bias (23).

We also developed a nomogram for predicting CBDS recurrence after choledocholithotomy. According to previous studies, a C-index of >0.7 indicates that the established nomogram has good accuracy and acceptable discriminatory power (31). The C-index of our developed nomogram was 0.758 (95% CI: 0.698–0.818), and the internal validation value based on the bootstrap method was 0.758 (95% CI: 0.641–0.875); these values demonstrated the effectiveness of the model.

The present study also has some limitations. First, the diagnosis and selection of patients were performed by clinicians; however, there still might be some unknown factors that could have influenced our model. Second, because of the retrospective nature of this single-center study, the sample size was limited, and clinical data were missing for a few patients; this introduced certain limitations and potential selection bias in the study. Third, the study conducted only internal validation of the model and currently lacks external data to further evaluate the predictive ability of the model.

## 5. Conclusion

In conclusion, because of the delayed recurrence of CBDS, it is recommended to continue follow-up of patients after choledocholithotomy. Our results indicated that a history of cholecystectomy, WBC count ≥11.0 × 10^9^/L, preoperative TBIL level ≥ 36.5 mmol/L, number of stones ≥2, maximum stone diameter ≥ 0.85 cm, and T-tube drainage were risk factors of CBDS recurrence after choledocholithotomy in elderly patients. We also developed a prediction model for CBDS recurrence with high efficiency and accuracy. We anticipate that our present research will contribute to improve the prognosis of patients with CBDS.

## Data availability statement

The original contributions presented in the study are included in the article/supplementary material, further inquiries can be directed to the corresponding author.

## Ethics statement

Ethical review and approval was not required for the study on human participants in accordance with the local legislation and institutional requirements. Written informed consent from the participants was not required to participate in this study in accordance with the national legislation and the institutional requirements.

## Author contributions

HW and Z-jL conception and design and drafting and revision of the manuscript. Y-qH and PT was responsible for tables and figures. S-yD, WZ, and S-yY helped with the literature gathering. All authors critically appraised the final manuscript, gave final approval of the version to be published, and agreed to be accountable for all aspects of the work in ensuring that questions related to the accuracy or integrity of any part of the work are appropriately investigated and resolved.
